# Supplementary material for: The genome sequence of the Scarce Hook-tip, Sabra harpagula (Esper, 1786) (Lepidoptera: Drepanidae)
Source: Wellcome Open Res. 2026 May 26;11:308. [Version 1] doi: 10.12688/wellcomeopenres.26632.1 (PMC13234554; doi:10.12688/wellcomeopenres.26632.1)
Supplement: Peer review history

# Reviewer report: Reviewer response for version 1

**DOI:** 10.21956/wellcomeopenres.29328.r156832

**Published:** 2026-06-05

**Authors:** Gulab D Khedkar

**Affiliations:** 1 Dr Babasaheb Ambedkar Marathwada University, Aurangabad, Maharashtra, India

**Data note** **title:** 

*The genome sequence of the Scarce Hook-tip, Sabra harpagula (Esper, 1786) (Lepidoptera: Drepanidae)*

**Peer** **review** **report**

 This manuscript presents a high-quality chromosome-level genome assembly for *Sabra harpagula*. The study provides the first high-quality genomic resource for the genus *Sabra* and represents a valuable contribution to the growing genomic resources available for Lepidoptera. The manuscript (Data Note) is well written, clearly organized, and provides sufficient biological background on the species. The sequencing strategy, assembly workflow, scaffolding, curation procedures, and quality assessment methods are comprehensive and follow current best practices in genome assembly. The authors provide detailed methodological descriptions, ensuring transparency and reproducibility.

 The assembly metrics are excellent, with near-complete chromosomal assignment, high BUSCO completeness, strong consensus quality values, and high k-mer completeness. The incorporation of PacBio HiFi sequencing, Hi-C scaffolding, and extensive quality-control analyses has resulted in a robust reference-quality genome assembly. Furthermore, the public availability of the raw sequencing data and assembly enhances the utility of this resource for the broader scientific community.

 I have only one minor suggestion. The manuscript could briefly expand on the potential applications of this genome resource for future studies of Drepanidae evolution, comparative genomics, adaptation, and conservation biology. However, this is optional and does not affect the scientific quality of the work.

 Overall, the manuscript is scientifically sound, technically rigorous, and provides an important genomic resource. I recommend the manuscript for indexing in its current form.

Are sufficient details of methods and materials provided to allow replication by others?

Yes

Is the rationale for creating the dataset(s) clearly described?

Yes

Are the datasets clearly presented in a useable and accessible format?

Yes

Are the protocols appropriate and is the work technically sound?

Yes

Reviewer Expertise:

Genomics, population genetics

I confirm that I have read this submission and believe that I have an appropriate level of expertise to confirm that it is of an acceptable scientific standard.

# Reviewer report: Reviewer response for version 1

**DOI:** 10.21956/wellcomeopenres.29328.r156824

**Published:** 2026-06-03

**Authors:** Fedor Čiampor

**Affiliations:** 1 Slovak Academy of Sciences, Bratislava, Slovakia

This data note presents a high-quality chromosome-level genome assembly for Sabra harpagula, the Scarce Hook-tip moth. The study is clearly structured and provides short background on the species, including its distribution, life cycle, host plant, and taxonomic placement. The methodology is detailed, covering sample collection, DNA barcoding, nucleic acid extraction, PacBio HiFi sequencing, Hi-C scaffolding, RNA sequencing, assembly, and quality assessment.

 The note is a concise but valuable. Its main contribution is the production of the first high-quality genome for the genus Sabra, adding an important resource for Lepidoptera genomics, biodiversity research, and future comparative studies.

 I have two comments on the manuscript. The first sentence of the "Background" section sounds as if this were a species found only in Britain (or at most in Europe, according to the following paragraph). Actually, it is apparently widespread throughout the Palearctic. I think a mention of this would be appropriate in the manuscript.

 Why is the purified DNA yield listed, but not the RNA yield?

Are sufficient details of methods and materials provided to allow replication by others?

Yes

Is the rationale for creating the dataset(s) clearly described?

Yes

Are the datasets clearly presented in a useable and accessible format?

Yes

Are the protocols appropriate and is the work technically sound?

Yes

Reviewer Expertise:

animal DNA metabarcoding

I confirm that I have read this submission and believe that I have an appropriate level of expertise to confirm that it is of an acceptable scientific standard.

# Reviewer report: Reviewer response for version 1

**DOI:** 10.21956/wellcomeopenres.29328.r156825

**Published:** 2026-06-02

**Authors:** Rajib Deb

**Affiliations:** 1 ICAR-National Research Centre, Guwahat, Assam, India

The manuscript presents a high-quality chromosome-level genome assembly for *Sabra harpagula* generated as part of the Darwin Tree of Life project. The rationale for dataset generation is clearly described, and the assembly provides a valuable genomic resource as the first high-quality genome for the genus *Sabra*.

 The sequencing, assembly, scaffolding, curation, and quality assessment protocols are appropriate and technically sound. The assembly achieves excellent quality metrics, including high BUSCO completeness, strong consensus quality, and near-complete chromosome assignment.

 Methods are described in sufficient detail to support reproducibility, with clear reporting of sample collection, laboratory procedures, software tools, and accession numbers. The raw data and assembled genome are publicly available and presented in a usable and accessible format.

 I have no major concerns. The manuscript is scientifically sound and suitable for indexing in its current form. Minor optional improvements could include additional discussion of future genome annotation plans and chromosome nomenclature, but these are not required for publication.

Are sufficient details of methods and materials provided to allow replication by others?

Yes

Is the rationale for creating the dataset(s) clearly described?

Yes

Are the datasets clearly presented in a useable and accessible format?

Yes

Are the protocols appropriate and is the work technically sound?

Yes

Reviewer Expertise:

Genomics

I confirm that I have read this submission and believe that I have an appropriate level of expertise to confirm that it is of an acceptable scientific standard.
